# Congenital Cataract in *Gpr161^vl/vl^* Mice Is Modified by Proximal Chromosome 15

**DOI:** 10.1371/journal.pone.0170724

**Published:** 2017-01-30

**Authors:** Bo I. Li, Myka R. Ababon, Paul G. Matteson, Yong Lin, Vikas Nanda, James H. Millonig

**Affiliations:** 1 Center for Advanced Biotechnology and Medicine, Rutgers University, Piscataway, New Jersey, United States of America; 2 Department of Neuroscience and Cell Biology, Rutgers University, Piscataway, New Jersey, United States of America; 3 Robert Wood Johnson Medical School, Rutgers University, Piscataway, New Jersey, United States of America; 4 Division of Biometrics, Rutgers Cancer Institute of New Jersey, Rutgers University, New Brunswick, New Jersey, United States of America; 5 Department of Biochemistry, Rutgers University, Piscataway, New Jersey, United States of America; 6 Department of Genetics, Rutgers University, Piscataway, New Jersey, United States of America; University of Iowa, UNITED STATES

## Abstract

The morphology and severity of human congenital cataract varies even among individuals with the same mutation, suggesting that genetic background modifies phenotypic penetrance. The spontaneous mouse mutant, *vacuolated lens* (*vl*), arose on the C3H/HeSnJ background. The mutation disrupts secondary lens fiber development by E16.5, leading to full penetrance of congenital cataract. The *vl* locus was mapped to a frameshift deletion in the orphan G protein-coupled receptor, *Gpr161*, which is expressed in differentiating lens fiber cells. When *Gpr161*^*vl/vl*^ C3H mice are crossed to MOLF/EiJ mice an unexpected rescue of cataract is observed, suggesting that MOLF modifiers affect cataract penetrance. Subsequent QTL analysis mapped three modifiers (*Modvl3-5*: **Mod**ifier of ***vl***) and in this study we characterized *Modvl4* (Chr15; LOD = 4.4). A *Modvl4*^MOLF^ congenic was generated and is sufficient to rescue congenital cataract and the lens fiber defect at E16.5. Additional phenotypic analysis on three subcongenic lines narrowed down the interval from 55 to 15Mb. In total only 18 protein-coding genes and 2 micro-RNAs are in this region. Fifteen of the 20 genes show detectable expression in the E16.5 eye. Subsequent expression studies in *Gpr161*^*vl/vl*^ and subcongenic E16.5 eyes, bioinformatics analysis of C3H/MOLF polymorphisms, and the biological relevancy of the genes in the interval identified three genes (*Cdh6*, *Ank* and *Trio*) that likely contribute to the rescue of the lens phenotype. These studies demonstrate that modification of the *Gpr161*^*vl/vl*^ cataract phenotype is likely due to genetic variants in at least one of three closely linked candidate genes on proximal Chr15.

## Introduction

Congenital cataract is the presence of an opacity in the lens at birth, affecting approximately 3 out of 10,000 live births in United States [1]. Similar to other birth defects, congenital cataract has a genetic basis with approximately 30–50% of the disorder being transmissible [2]. Several groups of genes are involved in congenital cataract, including lens crystallins (*Crya*, *Cryb* and *Cryg*), gap junction proteins (GJA3 and GJA8), membrane proteins (MIP), filament proteins (BFSP1 and BFSP2) and transcription factors (*Hsf4* and *Pax6*) [3, 4]. Human congenital cataract can be caused by autosomal dominant, autosomal recessive or X-linked mutations [5, 6]. However, the same genetic mutation in different families can affect the penetrance or severity of the congenital cataract, suggesting that additional genes or environmental factors can modify the primary mutation [3, 7, 8].

Mouse mutant models of congenital cataract have significantly contributed to our understanding of the disorder at a molecular level. Several genes that cause congenital cataract in mice also contribute to human cataracts, including *Cryg*, *Connexin*, *Foxe3* and *Sox1* [9, 10]. However, whether genetic variation can modify the penetrance and severity of mouse congenital cataract remains to be determined. In this study, we present the first multi-genic mouse model of congenital cataract.

To understand the causes of congenital cataract, we have been studying the *vacuolated lens (vl)* mouse mutant. The *vl* mutation arose spontaneously on the C3H/HeSnJ inbred background. Homozygous *vl/vl* C3H mice display congenital cataract with 100% penetrance. Of post-weaning adults, >95% show visible bilateral cataracts whereas the remaining show unilateral cataract. In addition, homozygous *vl/vl* mutants display neural tube defects (NTDs, 100% penetrance), embryonic lethality (~36% penetrance) and belly spot phenotype (<5% penetrance) [11].

The *vl* locus was positionally cloned to an 8bp deletion in the orphan G-protein coupled receptor, *Gpr161*. The deletion shifts the open reading frame, which truncates the receptor at the C-terminal intracellular domain. This truncation causes reduced steady state protein levels and a disruption of receptor mediated endocytosis, suggesting that the *Gpr161*^*vl*^ allele is a hypomorphic mutation. Consistent with the cataract phenotype, *Gpr161* is expressed throughout lens development. At E12.5 and E14.5, *Gpr161* transcripts are restricted to differentiating lens fiber cells and are absent from the proliferating anterior lens epithelium [11]. We previously determined that all the *Gpr161*^*vl/vl*^ embryos display normal lens phenotype up until E14.5. Starting at E14.5, when secondary lens fiber differentiation begins, a subset of *Gpr161*^*vl/vl*^ embryos exhibit abnormal lens fiber organization and vacuoles and by E16.5, 100% of the *Gpr161*^*vl/vl*^ embryos display a disorganized lens fiber phenotype.

Interestingly, genetic background modifies the *Gpr161*^*vl/vl*^ mutant phenotypes. When we previously crossed *Gpr161*^*vl/vl*^ C3H to the MOLF/EiJ genetic background, we found that the incidence of congenital cataract is decreased by 85%, along with a total rescue of embryonic lethality and 40% increased incidence of belly spot phenotype. These data suggested that unlinked modifiers could bypass the effect of the *Gpr161*^*vl/vl*^ mutation, establishing *Gpr161*^*vl*^ as a multi-genic mouse mutant model for congenital cataract.

Subsequent Quantitative Trait Locus (QTL) analysis mapped three modifiers from MOLF background: *Mod*ifiers of *vacuolated lens*—*Modvl3*, *4* and *5* [11, 12]. Previous work has characterized *Modvl3* (Chr4; LOD = 4.2) [11] and *Modvl5* (Chr18; LOD = 5.0) [13] and although *Modvl3* was mapped as a cataract modifier by QTL analysis, phenotypic analysis on *Modvl3*^MOLF^ and *Modvl5*^MOLF^ congenics found neither of these two loci is sufficient to modify the penetrance of congenital cataract ([13] and unpublished data).

In this study, we characterized *Modvl4* (Chr15; LOD = 4.4). Genotyping and morphological analyses demonstrated that *Modvl4*^MOLF^ partially rescues *Gpr161*^*vl/vl*^ congenital cataract. To further delimit the modifying region, three subcongenic lines were generated which narrowed down the interval to 18 protein-coding genes and 2 miRNAs. RTPCR analysis identified fifteen genes with detectable expression in E16.5 eye. We further investigated the likely contribution of these fifteen genes to the *Modvl4*^MOLF^ modifying effect by their biological relevancy to lens development or Gpr161 signaling, the presence of likely functional C3H/MOLF polymorphisms using online resequencing data for the inbred lines, and measuring mRNA levels in *Gpr161*^*vl/vl*^ eyes to determine whether the congenic rescued any expression difference. These studies identified three genes in the interval, *Cdh6* (Cadherin-6), *Ank* (pyrophosphate transporter) and *Trio* (Guanine exchange factor), that likely contribute to the *Modvl4*^*MOLF*^ cataract modifying effect.

## Results

A *Modvl4*^MOLF^ congenic was generated to test the sufficiency of this locus to rescue *Gpr161*^*vl/vl*^ phenotypes. C3H/MOLF hybrid mice were backcrossed to C3H isogenic mice for 8 generations and progeny were selected based on SSLP markers across the genome. The male mice with the most contribution to the C3H background but were still C3H/MOLF for the *Modvl4* QTL 95% CI were selected for further backcrossing until the congenic (*Modvl4*^*C/M*^) was generated (S1 Fig, panel A). *Modvl4*^*C/M*^ mice were then intercrossed to generate wild type *Modvl4*^M/M^ mice. Finally *Gpr161*^*+/vl*^ C3H mice were crossed to the wild type *Modvl4*^M/M^ to generate *Gpr161*^*+/vl*^*Modvl4*^*C/M*^, which were then crossed to *Gpr161*^*+/+*^*Modvl4*^*M/M*^ to produce *Gpr161*^*+/vl*^*Modvl4*^*M/M*^ progeny (S1 Fig, panel B).

### *Modvl4*^MOLF^ partially rescues *Gpr161*^*vl/vl*^-associated cataract in a dominant manner

**Fig 1:**
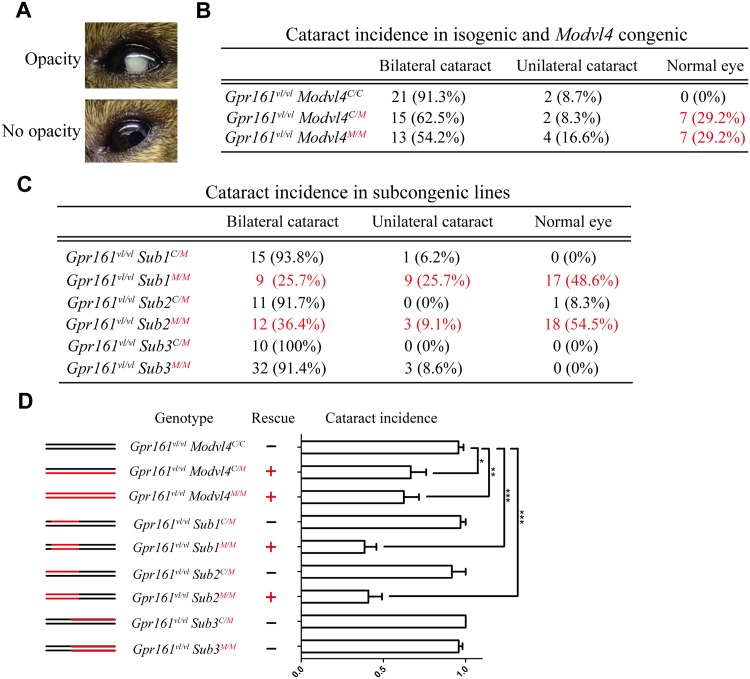
*Modvl4*^MOLF^ congenic and subcongenic lines rescue *Gpr161*^*vl/vl*^ cataracts. (A) Individual eyes from adult *Gpr161*^*vl/vl*^ *Modvl4*^*C/C*^, *Gpr161*^*vl/vl*^ *Modvl4*^*C/M*^ and *Gpr161*^*vl/vl*^ *Modvl4*^*M/M*^ mice were inspected under a stereomicroscope and the presence (top) or absence (bottom) of an opacity was recorded. (B) For C3H isogenic and *Modvl4* congenic littermates, the number and percentage of animals with bilateral cataract, unilateral cataract, and normal eyes are listed. (C) For the C/M and M/M alleles of each subcongenic lines, the number and percentage of animals with these lens phenotypes are listed. (D) The incidence of cataract for the different genotypes is quantified and compared to each other. The lines on the left illustrate the C3H (black) and MOLF (red) contribution to the *Modvl4* 95% CI for each congenic. Both *Gpr161*^*vl/vl*^ *Modvl4*^*C/M*^ and *Gpr161*^*vl/vl*^ *Modvl4*^*M/M*^ display a ~35% lower incidence of cataract when compared to *Gpr161*^*vl/vl*^ *Modvl4*^*C/C*^ isogenic mice. In addition, *Gpr161*^*vl/vl*^ *Sub1*^*M/M*^ and *Gpr161*^*vl/vl*^ *Sub2*^*M/M*^ display a ~60% lower incidence of cataract, which is also significantly lower than the *Gpr161*^*vl/vl*^ *Modvl4*^*C/C*^ isogenic mice. (*: P<0.05; **: P< 0.01; ***: P<0.001; All P values were calculated by one-way ANOVA with post-hoc Tukey’s multiplicity adjustment.—: no rescue; +: rescue)

To investigate if the *Modvl4*^MOLF^ congenic modifies the cataract phenotype, adult progeny of *Gpr161*^*vl/vl*^ *Modvl4*^*C/C*^, *Gpr161*^*vl/vl*^ *Modvl4*^*C/M*^ and *Gpr161*^*vl/vl*^ *Modvl4*^*M/M*^ littermates were generated from the two crosses: *Gpr161*^*+/vl*^ *Modvl4*^*C/C*^ x *Gpr161*^*+/vl*^ *Modvl4*^*C/M*^ and *Gpr161*^*+/vl*^ *Modvl4*^*M/M*^ x *Gpr161*^*+/vl*^ *Modvl4*^*M/M*^. Examination of eyes under a stereomicroscope determined the presence (Fig 1A top) or absence (Fig 1A bottom) of ocular opacities (also see S2 Fig for phenotypes of ten individual animals). For each genotype, the number of animals with bilateral cataract, unilateral cataract or normal eyes were determined (Fig 1B). In *Gpr161*^*vl/vl*^ *Modvl4*^*C/C*^, all animals display bilateral or unilateral cataract whereas in *Gpr161*^*vl/vl*^ *Modvl4*^*C/M*^ and *Gpr161*^*vl/vl*^ *Modvl4*^*M/M*^, about 30% of the animals display eyes that are grossly indistinguishable from wild type. To quantify this phenotype, each individual animal was assigned a value: 1 for bilateral cataract, 0.5 for unilateral cataract and 0 for normal. The average value represents the incidence of cataract for each genotype. Both *Gpr161*^*vl/vl*^ *Modvl4*^*C/M*^ and *Gpr161*^*vl/vl*^ *Modvl4*^*M/M*^ mice display significantly (~35%) lower incidence compared to *Gpr161*^*vl/vl*^ *Modvl4*^*C/C*^ (Fig 1D). We conclude that *Modvl4*^MOLF^ partially rescues *Gpr161*^*vl/vl*^-associated cataract in a dominant manner.

### Subcongenic analysis defines the *Modvl4* cataract modifier interval

According to the mouse genome (GRCm38/mm10 Assembly), more than 350 genes and ESTs are located in the *Modvl4* 95% CI, making it difficult to determine which genes contribute to the rescue of the cataract phenotype. To narrow down the interval, *Modvl4*^*C/M*^ congenic was further backcrossed to C3H isogenic mice. Subcongenic progeny with recombination breakpoints within the *Modvl4* 95% CI was selected by genotyping 14 SSLP markers spanning *Modvl4*. Three subcongenic lines were generated: two of them (*Sub1* and *Sub2*) contain the MOLF background for proximal *Modvl4* but differ in the proximal breakpoint, whereas the third line (*Sub3*) contains the MOLF background for distal *Modvl4* (S1 Fig, panels C and D). For each subcongenic line, two crosses were performed to generate adult progeny. To test the heterozygous rescuing effects of the congenic on cataract, the *Gpr161*^*+/vl*^ *Sub*^*C/C*^ x *Gpr161*^*+/vl*^ *Sub*^*M/M*^ (*Sub* = *Sub1*, *2* and *3*) mating was performed, while a *Gpr161*^*+/vl*^ *Sub*^*M/M*^ x *Gpr161*^*+/vl*^ *Sub*^*M/M*^ mating was performed to examine homozygous effects.

The effect of the subcongenics on the cataract phenotype was quantified using the same method mentioned above. For *Gpr161*^*vl/vl*^ *Sub1* and *Sub2* heterozygotes (*Gpr161*^*vl/vl*^ *Sub1*^*C/M*^, *Gpr161*^*vl/vl*^ *Sub2*^*C/M*^) as well as *Gpr161*^*vl/vl*^ *Sub3* heterozygotes and homozygotes (*Gpr161*^*vl/vl*^ *Sub3*^*C/M*^ and *Gpr161*^*vl/vl*^ *Sub3*^*M/M*^), no significant difference is observed when compared to the *Gpr161*^*vl/vl*^ *Modvl4*^C/C^ mice. However, for *Gpr161*^*vl/vl*^ *Sub1* and *Sub2* homozygotes (*Gpr161*^*vl/vl*^ *Sub1*^*M/M*^ and *Gpr161*^*vl/vl*^ *Sub2*^*M/M*^), the incidence of cataract is reduced by ~60%, and is significantly lower than *Gpr161*^*vl/vl*^ *Modvl4*^C/C^ (Fig 1C and 1D).

Since both the *Sub1* and *Sub2* homozygotes can rescue the cataract phenotype while the *Sub3*^*MOLF*^ congenic has no effect, these results indicate that the MOLF background within either *Sub1 or Sub2* is sufficient to partially rescue *Gpr161*^*vl/vl*^-associated cataract in a recessive manner. Because the *Sub1*^*MOLF*^ congenic is contained within *Sub2*^*MOLF*^ (S1 Fig, panels C and D), *Sub1* delimits the minimal region sufficient for *Modvl4*^*MOLF*^ cataract rescue. In addition, because the original *Modvl4*^*MOLF*^ congenic (which is equal to the sum of the subcongenics) rescues cataract in a dominant manner, it suggests that genetic modifiers in the *Sub3* interact with proximal *Sub1 and Sub2* modifiers to improve the rescuing effect from a recessive to dominant mode of inheritance.

While it would be interesting to identify the MOLF modifiers in *Sub3* that contribute to the complex inheritance pattern of cataract in *Gpr161*^*vl/vl*^ mutants, this is difficult because 95% (more than 300) of the *Modvl4* genes are located in the *Sub3*^*MOLF*^ interval. In addition *Sub3*^MOLF^ does not function independently and must interact with the proximal *Sub1*^*MOLF*^ *or Sub2*^*MOLF*^ region to rescue the cataract phenotype. Therefore, we decided to focus on the cataract repressors that are situated in the *Sub1*^*MOLF*^ *and Sub2*^*MOLF*^ region. Because *Sub1*^*MOLF*^ is smaller than *Sub2*^*MOLF*^ and has the same rescuing effect as *Sub2*^*MOLF*^, we decided to focus all subsequent analyses on *Sub1*^*MOLF*^.

### *Sub1*^MOLF^ partially rescues lens fiber defect during secondary lens fiber differentiation

Previous analysis determined that *Gpr161*^*vl/vl*^ lens is normal until E14.5. Starting from E14.5, lens fiber disorganization is observed and by E16.5, 100% of the *Gpr161*^*vl/vl*^ lenses are abnormal with disorganizations specifically localized to the posterior medial and nasal bow regions [11]. To test whether *Sub1*^MOLF^ rescues these defects during development, a histological analysis was performed at E16.5.

**Fig 2:**
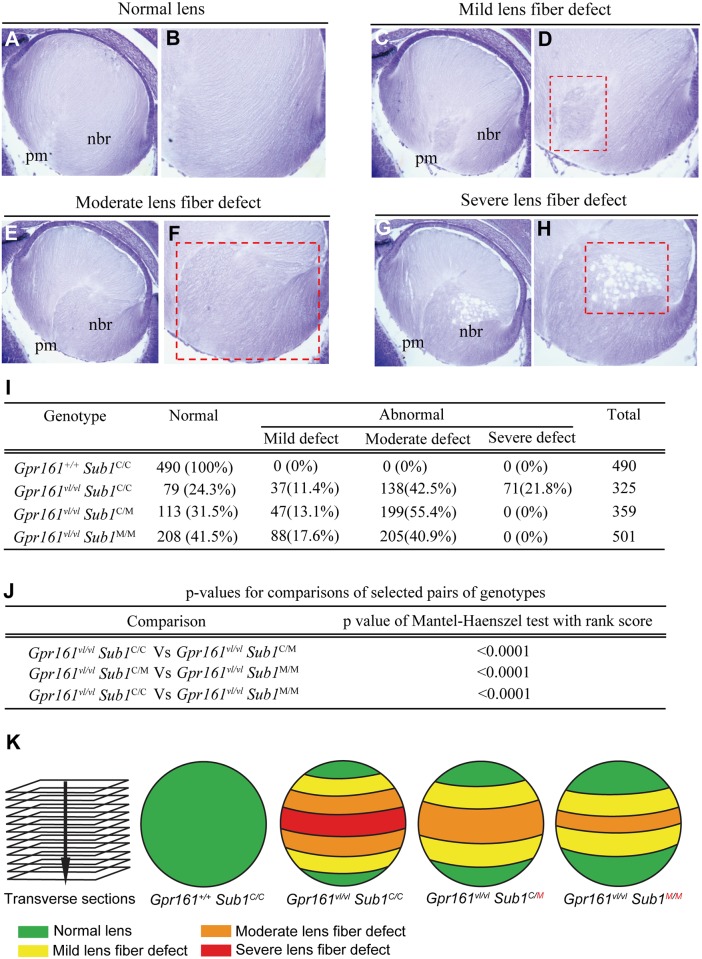
*Sub1*^*MOLF*^ congenic rescues *Gpr161*^*vl/vl*^ lens fiber defects. (A-H) Nissl stained transverse sections of E16.5 lens (A, C, E, G; 20X magnification), as well as magnified images of nasal bow and posterior medial region (B, D, F, H; 40X magnification) are shown (pm: posterior-medial region; nbr: nasal bow region). Normal (A, B) as well as mild (C, D), moderate (E, F) and severe (G, H) phenotypes are depicted. Red dashed boxes mark the region with abnormal lens fiber orientation and vacuoles. (I) The number and percentages (in parenthesis) of sections with the above phenotypes are shown for *Gpr161*^*+/+*^ *Sub1*^*C/C*^, *Gpr161*^*vl/vl*^ *Sub1*^*C/C*^, *Gpr161*^*vl/vl*^ *Sub1*^*C/M*^ and *Gpr161*^*vl/vl*^ *Sub1*^*M/M*^. (J) Pairwise comparisons between genotypes were performed using Mantel-Haenszel test with rank score. (K) The distribution of lens fiber phenotypes along the A-P axis is shown by representative illustrations for each of the four genotypes (green: normal; yellow: mild defect; orange: moderate defect and red: severe defect). In *Gpr161*^*vl/vl*^ mutant background, transverse sections that are closer to the lens equatorial region display more severe defects. In the *Modvl4*^*MOLF*^ congenic background, a partial rescue of the defect is observed by the reduction in severe lens fiber defect, and the expansion of normal and mild phenotypes.

**Fig 3:**
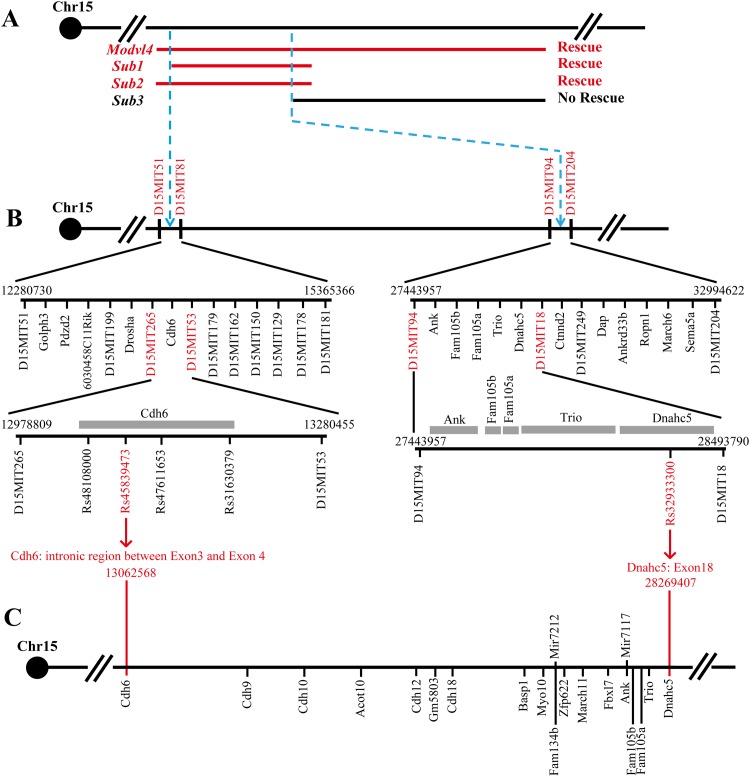
Fine mapping of *Modvl4*^*MOLF*^ 15 Mb minimal region. (A) The proximal extent of the minimal region that rescues the *Gpr161*^*vl/vl*^ cataract phenotype is defined by the proximal border of *Sub1* whereas the distal extent is defined by the proximal border of *Sub3* (blue dashed arrows; also refer to Fig 1C and 1D). (B) To determine the proximal and distal breakpoints of the 15 Mb cataract modifying region, SSLP and SNP genotyping was performed using genomic DNA sample from *Sub1* and *Sub3* congenic mice. Left: the proximal border was previously determined to be between D15MIT51 and D15MIT81 (blue dashed arrow). Further analysis delimited the proximal border to be between D15MIT265 and D15MIT53. Only one gene Cadherin 6 (*Cdh6)* is within this interval. Additional SNP genotyping determined that the proximal border is distal to rs45839473 (Chr15:13062568; between exon 3 and 4 of *Cdh6*). Right: the distal border was previously determined to be between D15MIT94 and D15MIT204 (blue dashed arrow). Additional SSLP and SNP markers further determined the border to be proximal to rs32933300 (Chr15:28269407; in exon 18 of *Dnahc5*). (C) The Rs45839473 SNP sits between Exon 3 and 4 of *Cdh6* while the Rs32933300 is within Exon 18 of *Dnahc5* (red arrows). In total 18 protein coding genes and 2 miRNAs are within the 15 Mb region flanked by the two SNPs (Chr15: 13062568–28269407). The genes are aligned across the chromosome based on their genetic loci. All labels of base pair information are based on mouse genome assembly GRCm38/mm10.

**Fig 4:**
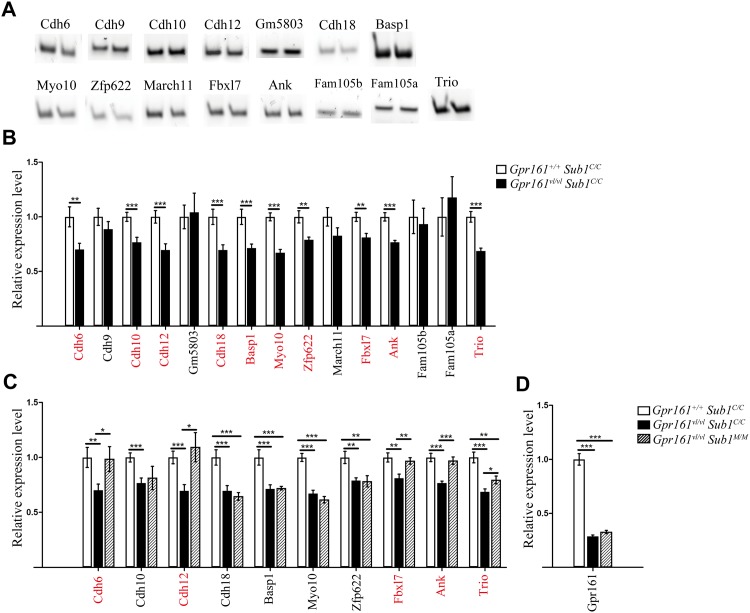
Expression of the candidate genes at E16.5 eye. (A) The *in vivo* expression of all 20 genes within the 15 Mb interval was assessed by performing RT-PCR using E16.5 eye cDNA. A total of 15 genes display detectable expression and PCR results are represented by gel electrophoresis (two biological replicates per gene). (B) QRT-PCR compared the expression level of the 15 genes and *Gpr161* between *Gpr161*^*+/+*^ *Sub1*^*C/C*^ and *Gpr161*^*vl/vl*^ *Sub1*^*C/C*^ E16.5 eyes. Ten of them (highlighted in red) showed reduced expression in *Gpr161*^*vl/vl*^ *Sub1*^*C/C*^. (C) QRT-PCR further compared the expression level of the 10 genes highlighted in (B) among *Gpr161*^*+/+*^ *Sub1*^*C/C*^, *Gpr161*^*vl/vl*^ *Sub1*^*C/C*^ and *Gpr161*^*vl/vl*^ *Sub1*^*M/M*^ E16.5 eyes. The expression level of 5 genes is fully (*Cdh6*, *Cdh12*, *Fbxl7* and *Ank*) or partially (*Trio*) restored by *Gpr161*^*vl/vl*^ *Sub1*^*M/M*^. (D) For *Gpr161*, a 70% reduced expression is observed in *Gpr161*^*vl/vl*^ *Sub1*^*C/C*^, which is not rescued by *Gpr161*^*vl/vl*^ *Sub1*^*M/M*^. All qRTPCR data in (B), (C) and (D) represent averages of six biological replicates per genotype. (*: P<0.05; **: P< 0.01; ***: P<0.001; Student’s t-test; two tailed, unpaired. P values were not adjusted for multiple testing)

A careful examination of all the serial transverse sections of E16.5 lenses (1675 sections in total from 14 *Gpr161*^*+/+*^ *Sub1*^*C/C*^, 13 *Gpr161*^*vl/vl*^ *Sub1*^*C/C*^, 11 *Gpr161*^*vl/vl*^ *Sub1*^*C/M*^ and 16 *Gpr161*^*vl/vl*^ *Sub1*^*M/M*^ lenses) revealed that the abnormal phenotypes can be categorized into four groups: 1) sections with **normal lens fiber**, which have the typical packing of differentiated lens fiber cells (Fig 2A and 2B); 2) sections with **mild lens fiber defects** which display disorganized lens fibers that are restricted only to the posterior medial region (Fig 2C and 2D); 3) sections with **moderate lens fiber defects** which have disorganized lens fibers in both the posterior medial and nasal bow regions of the lens (Fig 2E and 2F) and 4) sections with **severe lens fiber defects** that display lens fiber disorganization in posterior medial and nasal bow regions, as well as vacuoles in the nasal bow region (Fig 2G and 2H).

All 490 sections derived from 14 wild-type *Gpr161*^*+/+*^ *Sub1*^*C/C*^ lenses display normal lens fiber phenotypes. The 325 sections generated from 13 mutant *Gpr161*^*vl/vl*^ *Sub1*^*C/C*^ lenses, however, displayed all four phenotypic categories (Fig 2I and for individual lens, refer to S1 Table). Because serial sections were generated and placed in an ordered fashion on the microscopic slides, their relative position along the anterior-posterior axis can be estimated. We found that sections with more severe lens fiber defects are from the equatorial region of the lens, whereas sections with less severe defects are anterior or posterior of the equatorial region (see Fig 2K for illustration). These results suggest that there is an area of disorganized lens fibers in the center of the *Gpr161*^*vl/vl*^ lens, which could potentially be the precursor of the postnatal cataract phenotype.

The 359 sections from 11 *Gpr161*^*vl/vl*^ *Sub1*^*C/M*^ lenses and 501 sections from 16 *Gpr161*^*vl/vl*^ *Sub1*^*M/M*^ lenses were examined using the same criteria. Interestingly, no severe lens fiber defect is observed in either *Gpr161*^*vl/vl*^ *Sub1*^*C/M*^ or *Gpr161*^*vl/vl*^ *Sub1*^*M/M*^ lenses. Only normal, mild, and moderate phenotypes are detected. In addition, when compared to *Gpr161*^*vl/vl*^ *Sub1*^*C/C*^, a higher percentage of normal sections is observed (Fig 2I). In the congenics, sections with a moderate defect occupy the equatorial zone instead of sections with severe phenotypes as observed in *Gpr161*^*vl/vl*^ *Sub1*^*C/C*^. In addition, the moderate phenotype was sandwiched by sections with mild phenotype (Fig 2K). These results indicate that on the *Sub1*^*MOL*F^ congenic background, there is a less severe lens fiber defect, suggesting a partial rescue by *Sub1*^*MOLF*^ modifiers.

To investigate if the above differences among *Gpr161*^*vl/vl*^ *Sub1*^*C/C*^, *Gpr161*^*vl/vl*^ *Sub1*^*C/M*^ and *Gpr161*^*vl/vl*^ *Sub1*^*M/M*^ lenses are statistically significant, the frequencies for each of the four phenotypes were determined for each genotype. Pairwise comparisons among genotypes were made using Mantel-Haenszel test with non-parametric (rank) score [14]. Statistical significances were observed for all three comparison pairs (*Gpr161*^*vl/vl*^ *Sub1*^*C/C*^ vs *Gpr161*^*vl/vl*^ *Sub1*^*C/M*^; *Gpr161*^*vl/vl*^ *Sub1*^*C/M*^ vs *Gpr161*^*vl/vl*^ *Sub1*^*M/M*^ and *Gpr161*^*vl/vl*^ *Sub1*^*C/C*^ vs *Gpr161*^*vl/vl*^ *Sub1*^*M/M*^) (Fig 2J), indicating that *Sub1*^*MOLF*^ can partially suppress the severity of *Gpr161*^*vl/vl*^-associated lens fiber defect in a semi-dominant manner, confirming *Modvl4*^MOLF^ partially rescues lens defect in *Gpr161*^*vl/vl*^ during development.

### *Modvl4*^MOLF^ does not rescue *Gpr161*^*vl/vl*^-associated lethality

In addition to congenital cataract, we also examined the lethality phenotype in the *Modvl4*^*MOLF*^ congenic and subcongenics. We have previously determined that ~36% of the *Gpr161*^*vl/vl*^ mice on the C3H isogenic background die before weaning and other *Modvl* modifiers rescue the *vl*-associated lethality [13]. To test if *Modvl4*^MOLF^ also rescues *Gpr161*^*vl/vl*^-associated lethality, the *Gpr161*^*+/vl*^ *Modvl4*^*C/C*^ x *Gpr161*^*+/vl*^ *Modvl4*^*C/M*^ cross was performed and 291 adult progeny were genotyped. Taking into account the 36% lethality for the *Gpr161*^*vl/vl*^ mutation, *Gpr161*^*vl/vl*^ *Modvl4*^*C/C*^ and *Gpr161*^*vl/vl*^ *Modvl4*^*C/M*^ mice are expected to account for 8% of the total progeny. Chi-square test determined that for both genotypes, the observed numbers do not significantly deviate from the expected numbers (S2 Table, top section), consistent with the *Modvl4*^C/M^ congenic not rescuing lethality. To investigate whether being homozygous for the congenic affects rescue, *Gpr161*^*+/vl*^ *Modvl4*^*M/M*^ x *Gpr161*^*+/vl*^ *Modvl4*^*M/M*^ matings were performed and again no rescue of lethality was observed (S2 Table, bottom section). Similar mating strategies and statistical methods were also used to determine the lethality incidences on the three subcongenic backgrounds, and again no rescuing effect was observed (See S3 Table for details). Taken together, these results demonstrate that the *Modvl4*^MOLF^ congenic and subcongenics do not rescue the *Gpr161*^*vl/vl*^-associated lethality.

### Fine-mapping and annotation of the 15 Mb minimal region

Next we more precisely determined the recombination breakpoints for the minimal chromosomal interval that rescues the cataract phenotypes. Based on the previous genotyping results (S1 Fig, panel D), we expect the proximal border (marked by proximal border of *Sub1*) of the interval to be between D15MIT51 (Chr15: 12280730) and D15MIT81 (Chr15: 15365366), and the distal border (marked by proximal border of *Sub3*) to be between D15MIT94 (Chr15: 27443957) and D15MIT204 (Chr15: 32994622) (Fig 3A). To more precisely define the position of the recombination breakpoints, genomic DNA of the two subcongenic lines was genotyped for 11 additional SSLP and SNP markers. This analysis demonstrated that the proximal border is between Chr15: 13062568–13100445 while the distal border is between Chr15: 27443957–28269407. Therefore, the modifier interval that rescues the cataract phenotype is delimited to a 15 Mb region between Chr15:13062568 and Chr15:28269407 (Fig 3B).

The interval 15:13062568–28269407 was searched in UCSC Genome Browser (http://genome.ucsc.edu/cgi-bin/hgGateway; GRCm38/mm10 assembly) and Ensembl Genome Browser (http://www.ensembl.org; Build 75). Interestingly, a large part of the 15 Mb interval falls into a gene desert and only 20 genes are annotated for both genome browsers (Fig 3C; Also see Table 1 for descriptions of each gene).

**Table 1:** 20 genes within the 15Mb minimal modifier interval.

^a^Symbol	Category	Description of gene function
*Cdh6*	Protein coding	Cadherin 6; K-cadherin; Cell adhesion protein
*Cdh9*	Protein coding	Cadherin 9; T1-cadherin; Cell adhesion protein
*Cdh10*	Protein coding	Cadherin 10; T2-cadherin; Cell adhesion protein
*Acot10*	Protein coding	Acyl-coenzyme A thioesterase 10; Mitochondrial enzyme
*Cdh12*	Protein coding	Cadherin 12; Br-cadherin; Cell adhesion protein
*Gm5803*	Protein coding	Predicted gene 5803; Human ortholog is heterogeneous nuclear ribonucleoprotein A1-like 2
*Cdh18*	Protein coding	Cadherin 18; Cell adhesion protein
*Basp1*	Protein coding	Brain abundant, membrane attached signal protein 1
*Myo10*	Protein coding	Myosin X; Cytoskeleton reorganization, focal contacts formation and lamellipodial extension
*Fam134b*	Protein coding	Family with sequence similarity 134, member B; Endoplasmic reticulum-anchored autophagy receptor that mediates ER delivery into lysosomes
*Mir7212*	Micro-RNA	miRNA with unknown function
*Zfp622*	Protein coding	Zinc finger protein 622
*March11*	Protein coding	Membrane-associated ring finger (C3HC4) 11; E3 ubiquitin-protein ligase that mediates polyubiquitination of CD4
*Fbxl7*	Protein coding	F-box and leucine-rich repeat protein 7; Substrate recognition component of a E3 ubiquitin-protein ligase complex which mediates ubiquitination
*Ank*	Protein coding	Progressive ankylosis protein; Function as a transporter to regulate intra- and extracellular levels of inorganic pyrophosphate
*Mir7117*	Micro-RNA	miRNA with unknown function
*Fam105b*	Protein coding	OTU deubiquitinase with linear linkage specificity; Deubiquitinase that specifically removes linear ('Met-1'-linked) polyubiquitin chains from substrates
*Fam105a*	Protein coding	Family with sequence similarity 105, member A; Inactive ubiquitin thioesterase
*Trio*	Protein coding	Triple functional domain (PTPRF interacting); Guanine exchange factor that promote the exchange of GDP by GTP
*Dnahc5*	Protein coding	Dynein, axonemal, heavy chain 5; Subunit of microtubule-associated motor protein complex and functions as a force-generating protein with ATPase activity

^a^- From top to bottom: 20 genes within 15 Mb minimal interval are displayed based on their genetic loci on Chr15 (from proximal to distal).

### Identifying candidate QTGs and QTNs for the *Modvl4*^*MOLF*^ minimal region

Next we wanted to identify the candidate genes (QTGs) and genetic variations (QTNs) from the 20 genes that could be responsible for rescuing the lens fiber and cataract phenotypes. Four criteria were used:

#### Expression analysis

We would expect cataract QTGs to be expressed in the developing eye so the expression of the 20 genes were checked by RT-PCR using E16.5 eye cDNA from *Gpr161*^*+/+*^ *Sub1*^*C/C*^. Fifteen of the 20 genes show detectable expression (Fig 4A). No expression was observed for *Acot10*, *Fam134b*, *Mir7212*, *Mir7117* and *Dnahc5*, suggesting that they are not involved in the *Sub1*^*MOLF*^ rescue of the lens defects (data not shown).

#### Biological relevancy to lens development and Gpr161 signaling

Another criterion to identify candidate QTGs is the biological function of the 15 genes that display positive expression. Published literature was searched for their biological relevancy to lens development and pathways known to be downstream of Gpr161, including Shh, retinoic acid, and canonical Wnt signaling [13, 15]. Four of the genes regulate canonical Wnt signaling: *Cdh6*, *Basp1*, *Ank* and *Fam105b*. [16–20]. In addition, *Trio* is required for lens pit invagination [21] and is a guanine nucleotide exchange factor known to be downstream of GPCR signaling [22]. None of the 15 genes were found to be relevant to Shh or retinoic acid pathways.

#### SNPs/INDELs analysis

Candidate QTGs could also have coding SNPs/INDELs that affect the structure and function of the protein. Using a genetic variant query site for resequencing data of 28 mouse inbred strains, including C3H/HeJ and MOLF/EiJ, we searched the exons of the 15 genes that show positive expression in E16.5 eyes (http://www.sanger.ac.uk/sanger/Mouse_SnpViewer/rel-1410). A total of 258 exonic SNPs and 37 exonic INDELs were identified (S4 Table). 10 SNPs and 1 in-frame deletion are non-synonymous variations that affect amino acid sequences in five genes (*Cdh12*, *Basp1*, *Myo10*, *March11* and *Ank*).

To evaluate the functional impact of the 10 nsSNPs, online software was used. Polyphen (http://genetics.bwh.harvard.edu/pph2/) and SIFT (http://sift.jcvi.org/) were employed to predict impact of variants on protein function based on evolutionary conservation and available 3D crystal structures. Only V201A in Ank displayed positive results using both online tools (Table 2). Ank encodes a multipass transmembrane transporter that regulates intra- and extracellular concentrations of pyrophosphate (PPi) [23]. The protein is highly conserved across species with only 8 out of the 492 amino acids being different between human and mouse. The Valine allele in MOLF background is conserved from zebrafish to human, as well as among 24 out of the 28 inbred mouse strains, consistent with it being important in protein function. To determine the impact of the 15bp inframe deletion in *Myo10*, online software PROVEAN (http://provean.jcvi.org/index.php) was employed [24]. The five-amino acid deletion, SELAE/-, is predicted to be tolerant to protein structure (PROVEAN score = -0.137; Scores below -2.5 are considered deleterious) and consistently, it sits within a region that is only conserved among rodents. In conclusion, only *Ank* has one coding polymorphism that is likely to affect the structure and the function of the encoded protein.

**Table 2:** Non-synonymous SNPs of *Modvl4* candidate QTGs.

Gene	Position	SNP ID	Allele (C/M)	Amino acid substitution	^a^Polyphen score/prediction	^b^SIFT score/prediction
Cdh12	21237780	Rs31969979	G/C	Q34E	0.000/Benign	0.58/Tolerated
Cdh12	21520297	Rs216581334	C/T	P283S	0.001/Benign	0.27/Tolerated
Cdh12	21583865	Rs32879079	C/T	T597I	0.270/Benign	0.20/Tolerated
Cdh12	21583947	Rs32879078	G/T	M624I	0.000/Benign	1.00/Tolerated
Basp1	25364270	Rs224517210	C/T	A214T	Not in database	0.12/Tolerated
Myo10	25732087	Rs13482486	C/T	P350S	0.000/Benign	0.34/Tolerated
Myo10	25734027	Rs37304620	C/T	T367M	0.001/Benign	**0.05/Deleterious**
Myo10	25780409	Rs32166567	T/G	H850Q	0.000/Benign	0.29/Tolerated
March11	26409122	Rs32299178	C/T	P238L	**0.664/Possibly damaging**	0.26/Tolerated
Ank	27562809	Rs31625299	T/C	V201A	**0.932/Possibly damaging**	**0.00/Deleterious**

^a^- Polyphen PSIC score difference: generally, scores < 0.5 are considered benign and scores >0.5 are damaging (highlighted in bold).

^b^- SIFT: Amino acid substitutions with probabilities < 0.05 are predicted to be deleterious (highlighted in bold).

To model the structural and potential functional impact of V201A on Ank, we predicted its structure based on homology to known proteins using TMHMM server. Ank is projected to contain eight transmembrane-spanning helices (S3 Fig, panel A). The V201A substitution is situated within the fourth transmembrane helix, and interestingly sits in a groove that is known to mediate helix-helix interactions in the membrane (S3 Fig, panel B) [25–27]. Typically these motifs are of the form *AxxxBC* where *A* and *B* are small amino acids (Gly, Ser, Ala) that are flanked by *C*, a beta-branched amino acid (Val, Ile) [25]. The Val to Ala substitution at 201 alters the beta-branched nature of this flanking sidechain, potentially affecting Ank folding or protein-protein interactions between Ank and other binding partners in the membrane.

#### Sub1 qRTPCR analysis

Finally we also quantified the expression levels of the 15 genes in E16.5 eyes from *Gpr161*^*+/+*^ *Sub1*^*C/C*^, *Gpr161*^*vl/vl*^ *Sub1*^*C/C*^ and *Gpr161*^*vl/vl*^ *Sub1*^*M/M*^. Ten of the 15 genes display significantly reduced expression in *Gpr161*^*vl/vl*^ *Sub1*^*C/C*^ compared to *Gpr161*^*+/+*^ *Sub1*^*C/C*^, coinciding with the lens fiber defects appeared at this stage of development (Fig 4B). Interestingly, when the expression level of those ten genes were further measured in *Gpr161*^*vl/vl*^ *Sub1*^*M/M*^, which partially rescues lens fiber phenotypes, five genes display restored expression levels with 4 of them (*Cdh6*, *Cdh12*, *Fbxl7* and *Ank*) show wild type-like expression and for *Trio*, a partial restoration of expression is observed (Fig 4C). We conclude that the expression levels of *Cdh6*, *Cdh12*, *Fbxl7*, *Ank* and *Trio* are correlated to the presence of cataract in *Gpr161*^*vl/vl*^ *Sub1*^C/C^, and the rescue of cataract in the congenic background (Table 3 second column).

**Table 3:** Summary of *Modvl4* candidate QTGs.

Gene	^a^Altered expression level in *vl* and the congenic	^b^Presence of functional coding variations	^c^Biological relevancy to eye function
^d^**Cdh6**	Yes	No	Yes
Cdh9	No	No	No
Cdh10	No	No	No
Cdh12	Yes	No	No
Gm5803	No	No	No
Cdh18	No	No	No
Basp1	No	No	Yes
Myo10	No	No	No
Zfp622	No	No	No
March11	No	No	No
Fbxl7	Yes	No	No
^d^**Ank**	Yes	Yes	Yes
Fam105b	No	No	Yes
Fam105a	No	No	No
^d^**Trio**	Yes	No	Yes

^a^- QRTPCR determined the expression level of the fifteen candidate genes in the minimal interval using *Gpr161*^*+/+*^ *Sub1*^*C/C*^, *Gpr161*^*vl/vl*^ *Sub1*^*C/C*^ and *Gpr161*^*vl/vl*^ *Sub1*^*M/M*^ E16.5 eye cDNA. Five genes, *Cdh6*, *Cdh12*, *Fbxl7*, *Ank* and *Trio*, showed reduced expression in *Gpr161*^*vl/vl*^ *Sub1*^*C/C*^ but restored expression in *Gpr161*^*vl/vl*^ *Sub1*^*M/M*^. Whole genome C3H and MOLF sequencing data identified multiple C3H/MOLF variants in flanking and intronic sequence for all five genes that are predicted to affect transcription factor binding and may affect expression (see S5 Table for list of candidate non-coding SNPs and Indels).

^b^- Protein coding C3H/MOLF variants were identified from whole genome sequencing data and their predicted effect on protein structure and function by online software PolyPhen and SIFT is denoted.

^c^- Published literature was searched for genes that are relevant to lens development and Gpr161 downstream signaling.

^d^- Genes that fulfill at least two criteria are considered as candidate QTGs for the *Modvl4* modifying effect and are highlighted in bold.

Next, we investigated if genetic variation exists in the non-coding regions of these five genes that could explain the expression differences between *Gpr161*^*vl/vl*^ *Sub1*^C/C^ and *Gpr161*^*vl/vl*^ *Sub1*^M/M^. Flanking sequences (up to 50kb 5’ and 3’ of the gene) and intronic sequences were searched for all five genes (http://www.sanger.ac.uk/sanger/Mouse_SnpViewer/rel-1410) and a total of 129 Indels and SNVs were identified (S5 Table). We then investigated if any of these variants fall within predicted transcription factor binding sites by using online resources (http://www.gene-regulation.com/pub/programs/alibaba2/index.html). By comparing the results between C3H and MOLF, all genes were found to have multiple variants (89 total) that are predicted to affect transcription factor binding. We conclude that MOLF variants exist within 50kb of *Cdh6*, *Cdh12*, *Fbxl7*, *Ank* and *Trio* that could explain the expression differences between *Gpr161*^*vl/vl*^ *Sub1*^C/C^ and *Gpr161*^*vl/vl*^ *Sub1*^M/M^.

Lastly, we also measured *Gpr161* expression using E16.5 eye cDNA. The expression level dropped by 70% in *Gpr161*^*vl/vl*^ *Sub1*^*C/C*^ but is not rescued in *Gpr161*^*vl/vl*^ *Sub1*^*M/M*^ (Fig 4D). This result indicates that *Sub1*^MOLF^ is a bypass allele that rescues cataract by either acting through a parallel pathway, or restoring downstream signaling activities of *Gpr161*, compensating for the loss of *Gpr161* function in *Gpr161*^*vl/vl*^.

#### Combining all criteria to identify candidate QTGs

The Complex Trait Consortium (CTC) has established eight criteria for identification of candidate QTG [28]. The four criteria we described above belong to three of those eight criteria: 1) expression in the appropriate target tissue(s) or cell type(s); 2) polymorphisms in coding or regulatory regions and 3) published *in vitro*/*in vivo* functional studies. Generally, CTC requests at least two criteria to be fulfilled in order to consider a gene as QTG [28]. Among all 15 genes that are expressed in developing eyes, a total of 3 genes (*Cdh6*, *Ank* and *Trio*) have fulfilled at least two criteria (Table 3). Although this does not rule out the possibility that the other 12 genes may also contribute to the rescue of lens phenotypes by *Sub1*^*MOLF*^, we conclude that these three genes have the strongest evidence for contributing to the rescue of *Gpr161*^*vl/vl*^ cataract phenotype.

## Discussion

Phenotypic variability is commonly observed for human disease, affecting the penetrance and severity of most disorders [29–33]. For congenital cataract, studies have shown that the same mutation can result in differences in the morphology, location, color and density of cataracts. These results suggest that unlinked genetic variants can contribute to the modification of the primary mutation. While the influence of genetic background on the penetrance and expressivity of human congenital cataract has been postulated, how this may occur has not been investigated previously [3, 7, 8]. In this study, we took advantage of the natural genetic variations among different inbred mouse strains to model how unlinked modifiers could affect a primary mutation and affect the penetrance of congenital cataract.

*Gpr161*^*vl*^ is a unique polygenic mouse model for congenital cataract. We previously determined that the cataract incidence in *Gpr161*^*vl/vl*^ is variable among different inbred strains, with 100% penetrance on the C3H/HeSnJ background but only a 15% penetrance when crossed to the MOLF/EiJ background [11, 12]. In this study, we focused on the *Modvl4*^*MOLF*^ locus and by generating congenic and subcongenics for *Modvl4*^*MOLF*^, we identified a 15Mb interval on proximal Chromosome 15 that is sufficient to partially restore normal lens fiber development. Three genes (*Cdh6*, *Ank* and *Trio*) were identified as candidate QTGs with coding and non-coding QTNs that are predicted to be functional. Our studies suggest that at least one of those QTGs and QTNs can affect the penetrance of the *Gpr161*^*vl*^ mutation, providing insight into how human congenital cataract may be modified by genetic background. In addition, since some of these genes have not been implicated in lens development previously, their further characterization may also provide new insight into the molecular basis of lens development and cataract.

### *Modvl4*^MOLF^ as a modifier of congenital cataract

Our previous congenic analysis for a different QTL, *Modvl5*^MOLF^ (Chr18; LOD = 5.0), determined that *Modvl5*^MOLF^ specifically rescues the lethality and neural tube defects (NTDs) associated with the *Gpr161*^*vl/vl*^ mutation, but did not affect congenic cataract [13]. Interestingly, we show in this study that *Modvl4*^MOLF^ has no effect on the lethality but instead partially rescues the cataract and lens fiber phenotypes. While it remains to be determined whether *Modvl4* also plays a role in modifying the NTD phenotypes, these two studies indicate that the pleiotropic effects of the *Gpr161*^*vl/vl*^ mutation on lethality and lens development are mediated by both *Gpr161* and unlinked genes situated on different chromosomes. By generating a *Modvl4*-*Modvl5*^MOLF^ double congenic, it will be interesting to investigate whether *Modvl4* and *5* act independently or play synergistic roles in regulating these *Gpr161*^*vl/vl*^ mutant phenotypes.

To narrow down the modifying interval of *Modvl4*, we generated three subcongenic lines for morphological analysis. Interestingly, our subcongenic studies revealed an unexpected, complicated inheritance pattern of the cataract modifying effect. We determined that the whole region of *Modvl4*^MOLF^ represses cataract in a dominant manner, whereas *Sub1* and *Sub2*, which have the MOLF background in the proximal portion of *Modvl4*, repress cataract in a recessive manner. *Sub3*, which has MOLF background in the distal portion of *Modvl4*, has no effect. These results indicate that while the QTGs in proximal *Modvl4* (*Sub1 and Sub2*) are sufficient to rescue cataract, additional QTGs in distal *Modvl4* (*Sub3*) genetically interact with proximal QTGs to improve the rescuing effect from a recessive to dominant mode of inheritance.

Within the scope of this study, we focused on proximal *Modvl4* that overlap in large part with the *Sub1* interval. Future work beyond the scope of this manuscript will investigate QTGs from the distal region that contributes to the complex inheritance pattern of the cataract modifying effect. As distal *Modvl4* interacts with proximal *Modvl4*, one hypothesis would be QTGs from distal region share similar functions as proximal QTGs to regulate lens development. For example, as our analysis identified the cell adhesion molecule *Cdh6* as a candidate QTG from *Sub1*, we might expect some QTGs in the distal *Modvl4* to be involved in cell adhesion. One potential candidate in the distal region is Cadherin-Associated Protein, Delta 2 (*Ctnnd2*), which forms a complex with cadherins to mediate intercellular adhesion [34, 35].

### E16.5 lens histological analysis revealed a partial rescue of lens fiber defect

Interestingly, although 30–50% of the adult eyes for the *Modvl4* congenic and subcongenic animals have no visible opacity, our histological analysis on E16.5 lens sections revealed that almost all (26 out of the 27 lens analyzed, refer to S1 Table) of the *Gpr161*^*vl/vl*^ *Sub1*^C/M^ and *Gpr161*^*vl/vl*^ *Sub1*^M/M^ lens display different severities of abnormal lens fiber phenotypes. The severity can be scored based on the size of affected area and the presence/absence of vacuoles in the nasal bow region. A careful inspection of all transverse sections along the A-P axis of each individual lens demonstrates that *Gpr161*^*vl/vl*^ *Sub1*^C/M^ lens has a decreased number of sections with severe and moderate defects and a greater number of sections with mild and normal phenotypes, compared to the *Gpr161*^*vl/vl*^ *Sub1*^C/C^ littermates. In addition, *Gpr161*^*vl/vl*^ *Sub1*^M/M^ embryos show a further shift from severe to normal phenotypes, compared to *Gpr161*^*vl/vl*^ *Sub1*^C/M^ embryos. These results indicate that the congenic mice display a smaller area of lens fiber abnormality compared to the C3H isogenic mutant mice.

Adult lens fiber disorganization is associated with lens opacity, reduced transmission of light through the lens, and decreased sight. In the congenic background, a less severe lens fiber defect diminishes the incidence of opacity. This would improve the transmission of light and the ability to see, although it remains to be determined if actual improvement of visual ability is achieved in the congenic. In addition, being heterozygous or homozygous for the *Sub1*^*MOLF*^ interval can repress the lens fiber defect during development, but only the *Sub1*^M/M^ subcongenic rescues congenital cataract in the adult, suggesting that there is a threshold in the lens fiber defect that determines the presence of lens opacity.

### Identifying candidate QTGs/QTNs from the 15 Mb interval of proximal Chr15

Analysis of our three subcongenic lines narrowed down the modifying interval from a 55Mb region with more than 350 genes/ESTs to a 15Mb region flanked by Rs45839473 and Rs32933300 that contains only 20 genes. Our expression analysis identified 15 genes with detectable mRNA in the developing eye. While it remains formally possible that the other 5 unexpressed genes may affect the Gpr161^*vl/vl*^ lens phenotype through a non-cell autonomous mechanism or by being expressed earlier in development, we decided to focus on the 15 expressed genes. To further narrow down the 15 genes, three criteria were used: 1) whether the expression level of the gene at E16.5 eye is disrupted in *Gpr161*^*vl/vl*^ and restored by the congenic, consistent with flanking/intronic C3H/MOLF variants regulating mRNA levels; 2) whether the gene has coding nonsynonymous variations that are predicted to affect the protein structure and 3) whether published manuscripts have determined that the gene has relevancy to lens development or Gpr161 downstream signaling. In total, we identified three genes (*Cdh6*, *Ank* and *Trio*) that fulfilled at least two criteria, and are considered as candidate QTGs of *Modvl4* according to the rationale established by Complex Trait Consortium [28].

This analysis has also identified candidate genes that could function during lens development and Gpr161 signaling. For instance, Trio is a Guanine Exchange Factor (GEF) so it may regulate G protein signaling downstream of Gpr161 in the lens. Interestingly, Trio also regulates lens pit invagination during development [21]. Cadherins are important for many different steps in lens development. For instance a switch from E- to N-cadherin expression is needed for secondary lens fiber differentiation, which is the stage when the *Gpr161*^*vl/vl*^ mutation affects lens development [36]. *Cdh6* also regulates axon-targeting in the visual circuit, and differentiation of retinal ganglion cells, amacrine cells, and photoreceptors, were disrupted in *Cdh6* zebrafish morphant [37, 38]. The identification of *Cdh6* as a *Modvl4*^*MOLF*^ QTG suggests that this cadherin may also be important for lens development. Finally, Ank is a membrane transporter for pyrophosphate (PPi). Pyrophosphate is generated during the hydrolysis of ATP to AMP. Maintenance of extracellular and intracellular concentrations of pyrophosphate is needed for normal articular cartilage cellular function [39]. In addition, PPi has also been shown to be a signaling molecule important for the generation of inositol pyrophosphates which regulate numerous processes including metabolic homeostasis and apoptosis [40]. The identification of Ank as a candidate QTG for *Modvl4* suggests that PPi levels and/or signaling through PPi may be important for lens development.

In summary, using *Modvl4*^MOLF^ congenic as a model, we studied the multigenic basis of *Gpr161*^*vl/vl*^-associated congenital cataract. We demonstrated that a 15Mb interval in proximal *Modvl4*^MOLF^ genetically interacts with *Gpr161* to partially rescue the lens fiber orientation defect and congenital cataract. Among all 20 genes situated in this region, we determined three genes (*Cdh6*, *Ank* and *Trio*) as candidate QTGs by multiple criteria. This study provides new insight into the multigenic basis of congenital cataract and identified novel candidate genes for future investigation.

## Materials and Methods

### *Modvl4*^*MOLF*^ congenic analysis

This study was carried out in strict accordance with the recommendations in the Guide for the Care and Use of Laboratory Animals of the National Institutes of Health. All the rodents used were approved by the IACUC of Rutgers University—Robert Wood Johnson Medical School (Protocol number is I12-113). Pregnant females and adult mice of either sex were anesthetized by intraperitoneal injection of Euthasol (Nembutal: Phenytoin solution; 200mg/kg), followed by cervical dislocation.

*Modvl4*^MOLF^ congenic mice were generated by backcrossing C3H/MOLF hybrid to C3H isogenic background for 8 generations (for detailed description, refer to S1 Fig, panel A). Progeny of each generation were genotyped for 65 SSLP markers that span the genome as described in [11].

The *Gpr161*^*+/vl*^ *Modvl4*^*C/M*^ x *Gpr161*^*+/vl*^ *Modvl4*^*C/C*^ and *Gpr161*^*+/vl*^ *Modvl4*^*M/M*^ x *Gpr161*^*+/vl*^ *Modvl4*^*M/M*^ matings were performed to generate adult progeny. Tail samples of post-weaning adults were collected and processed with Wizard SV genomic DNA purification kit (Promega). The DNA samples were genotyped for *Gpr161* and *Modvl4* 95% CI (PCR condition: 94°C 15 s, 51°C 15 s, 72°C 45 s-30 cycles). For *Gpr161*^*vl/vl*^ mutation, a primer pair flanking the 8-bp deletion as described in [11] was used. For *Modvl4*, 14 SSLP MIT markers that are evenly distributed within the *Modvl4* 55 Mb interval (from proximal to distal: D15MIT51, D15MIT81, D15MIT130, D15MIT252, D15MIT267, D15MIT94, D15MIT204, D15MIT49, D15MIT24, D15MIT86, D15MIT59, D15MIT195, D15MIT27 and D15MIT257) were genotyped (primer sequences are listed at genome.ucsc.edu). To test the effect of the *Modvl4*^MOLF^ on *Gpr161*^*vl/vl*^-associated lethality, the number of progeny of different genotypes were determined and compared to expected number using a Chi-square test as described in [13]. To test the effect of the *Modvl4*^MOLF^ congenic on congenital cataracts, adult *Gpr161*^*vl/vl*^ progeny derived from the two matings listed above were inspected for the presence or absence of opacity in the eye. Mice were sacrificed by cervical dislocation and placed under a stereomicroscope (Nikon SMZ800) for phenotypic inspection (5X magnification). Pictures of the left and right eyes were then taken by digital camera (SPOT RT Color).

### *Modvl4*^*MOLF*^ subcongenic analysis

To generate subcongenic mice, the *Gpr161*^*+/vl*^ *Modvl4*^*C/M*^ x *Gpr161*^*+/vl*^ *Modvl4*^*C/C*^ mating was performed. Progeny were genotyped for the 14 SSLP markers described above as well as for the *Gpr161*^*vl*^ mutation. *Gpr161*^*+/+*^ or *Gpr161*^*+/vl*^ animals with at least one recombination breakpoint within *Modvl4*^MOLF^ were used as founders to generate subcongenic lines (S1 Fig, panels C and D). Lethality and cataract phenotypes were tested in the subcongenic lines as described above.

### Histological analysis on the developing lens

Heads from E16.5 embryos were fixed overnight with Bouin’s Solution (Sigma) and stored in 70% EtOH at 4°C. The tissue was then dehydrated, embedded in paraffin, and transversely sectioned by rotary microtome (Leica RM2135, 10 μm in thickness). Serial paraffin sections were sequentially aligned on glass slides according to their location along the A-P axis. The paraffin sections were then processed using a standard Nissl staining protocol. Sections were inspected under the microscope (Nikon Eclipse E600) for quantification of the lens fiber defects. To determine the statistical significance of the rescuing effect of the congenics, the number of sections with different severities of lens fiber defects was compared in pairs using Mantel-Haenszel test with non-parametric (rank) score [14].

### Determination of recombination breakpoints for the 15Mb region

The cataract rescuing effect was mapped to a ~15Mb region with the proximal border (marked by proximal border of *Sub1*) being between D15MIT51 (Chr15: 12280730) and D15MIT81 (Chr15: 15365366), and the distal border (marked by proximal border of *Sub3*) being between D15MIT94 (Chr15: 27443957) and D15MIT204 (Chr15: 32994622). Therefore, genomic DNA of *Sub1* and *Sub3* were genotyped with additional SSLP and SNP markers. The following SSLP markers were used: proximal: D15MIT199, D15MIT265 and D15MIT53; Distal: D15MIT45, D15MIT163, D15MIT18 and D15MIT21. Primer sequences were obtained from Mouse Genome Browser Gateway (Assembly NCBI37/mm9).

After the break points were mapped between the two adjacent SSLP markers, SNPs were selected within the region for additional fine mapping. Genomic sequences spanning the SNP loci were PCR amplified and sequenced to determine whether C3H or MOLF allele of the SNP is present. The following primers were used: Rs48108000: F: GGAGAACCCCTCACGGAATAGTG; R: CCAAAGCCCCCAGTCTGATTG. Rs45839473: F: TATCGTGCTGGGACTTGAGACG; R: TTTGTTTTGGCGTGGGCTG. Rs47611653: F: AGGTGAATGAGAGAGGAGAGGAAAC; R: GGAAGGTGACAAATGATAGTTGGG. Rs31630379: F: GAGAGGAGGACTTTGTTACAGAGGC; R: GGCGTTTGGATTTGAACCG. Rs32933300: F: TGTGCTCTTTCTGCTCTTCCTGAC; R: AAATGACATCTCCCCCTCACCC.

### Gene expression analysis

RNA was extracted from E16.5 eyes using standard phenol-chloroform extraction method. The pellet was resuspended into 10ul nuclease-free water. 20ul of cDNA was generated from 4ul of RNA using SuperScript^™^ II Reverse Transcriptase (Invitrogen). For RT-PCR, 2ul of 1:10 diluted cDNA was used to amplify coding region of each gene (PCR condition: 94°C 30 s, 57°C 30 s, 72°C 45 s-30 cycles). 10ul of the PCR products were gel separated by 8% polyacrylamide gel electrophoresis.

QRT-PCR was performed by using ABI7900HT (Applied Biosystem). PCR condition: 94°C 30 s, 60°C 30 s, 72°C 40 s-40 cycles. Cycle Threshold (Ct) value was plotted against a standard curve to convert to relative gene expression level. The expression level was normalized to GAPDH level. Statistical analysis was performed using unpaired, two-tailed Student’s t-test.

The following primers were used for both RT-PCR and qRT-PCR: *Cdh6*: F: ATGACAACCCACCCCGTTTC; R: CCGCATTTTCTCCCACATCG. *Cdh9*: F: AAGTTCTTCTTTGAGCCAGTGCC; R: GGTAGGTGTTCATTTTGTTGCGG. *Cdh10*: F: CAGGTGGTTATTCAAGCCAAGG; R: CCAACTGGAGAGGATTCAAGAACTC. *Acot10*: F: AAATCACAGAAAGTCCTACCACCG; R: CAAGGTCCTCAAGAATCCTGCC. *Cdh12*: F: CAGTGCCAGAGTTGTTTACAGCATC; R: TGTCTTTCGCTTGAATGAGGACC. *Cdh18*: F: TGTCTCCGTGGGTATTAGAGTTCTG; R: AGTGATGGTGTGGATAACCTGACC. *Basp1*: F: GGGCTACAATGTGAACGACGAG; R: TCTCCTCCGTGCTCTCCTTGAC. *Myo10*: TTGCCAACGAATGCTATCGC; R: CTGATGACGGACAGGAACTTGAG. *March11*: F: GCCACAGACATTGAAGAAAGCAG; R: TGAGGTTAGTTGCGTTGGGTG. *Zfp622*: F: TGCCATCCCAATAACAGACTGC; R: TGCCAACACCAACTTTCTCTCC. *Fbxl7*: F: CATACACCAACCAAAGCCCAGAG; R: ATGAAGATGTGGACGAACCCC. *Ank*: F: ATGTGGATGAGTCTGTGGGGAG; R: TGGCTACGAAAACAACCTGAGC. *Trio*: F: TGACAGAATACGGCAGGAGGAC; R: ATCTTCAGCAGCGGCTTGATGG. *Dnahc5*: F: TGTTGGTGGACTCCGTCATCTC; R: CTTGGGCTTTTTCATCTTTCCG. *Gm5803*: F: TAAGTCCGAGTCTCCCAAGGA; R: TCAGACTCTTGTCGGTTGTTTGA. *Fam134b*: F: GCCAGCAAAACACCGCTGA; R: TAGCCGGGCATCCTTGTGT. *Fam105a*: F: TTCCACACCGTGGGTACTTG; R: GGGGTAGCAGACTGCAAAGT. *Fam105b*: F: ACTACCCGGCATCCTAACTCT; R: GCTCAGTAGGTGGTGTTGGG. *Gpr161*: F: CAGGCTTCAGCTACTCTCAGGATTC; R: CCTCTTTGATCTGTTCCACTTCGTC; *GAPDH*: F: TGTTCCTACCCCCAATGTGTCC; R: GGAGTTGCTGTTGAAGTCGCAG. For *Mir7212* and *Mir7117*, TaqMan probes were used for qRT-PCR (Assay IDs are 467080_mat for *Mir7212* and 466575_mat for *Mir7117*).

### Modeling membrane topology and structure of Ank

In this study, the mouse Ankylosis protein sequence was submitted to the TMHMM server version 2.0 (http://www.cbs.dtu.dk/services/TMHMM/) to identify trans-membrane domains. Using TMHMM, eight trans-membrane domains were predicted, each having computed membrane spanning confidence scores > 0.5: residues 86–106, 131–152, 158–180, 191–213, 327–346, 361–383, 403–423, 430–452. A previous published study predicted Ank to contain twelve transmembrane domains, using the TMpred prediction program [41]. We have opted to use TMHMM since it is considered to provide more true positive identifications and fewer false positives and false negatives than TMpred [42].

In our modeling using TMHMM, the V201A mutation lies in the center of the fourth predicted membrane-spanning domain. The length of membrane spanning domains (~20 residues) is consistent with a polytopic alpha-helical protein. The fourth domain (residues 191–213) was modeled as an alpha-helix using standard amino acid conformations in the molecular visualization platform pyMol (Version 1.7.0.3 Schrödinger, LLC).

### Identification of coding and non-coding genetic variants of candidate genes

The Mouse Genome Project Team at Wellcome Trust Sanger Institute has used next-generation sequencing to sequence key laboratory mouse strains including C3H/HeJ and MOLF/EiJ, and the data were uploaded to a query website (http://www.sanger.ac.uk/sanger/Mouse_SnpViewer/rel-1410). For identification of coding genetic variants, the minimal cataract modifying interval (Chr15: 13062568–28269407) as determined by subcongenic analysis was searched in the query site. Results were exported as a spreadsheet and C3H/MOLF genetic variants for the fifteen genes that are expressed in E16.5 eyes were selected (258 SNPs and 37 INDELs). 10 SNPs and 1 Indel were found to affect the amino-acid sequence of five genes (*Cdh12*, *Basp1*, *Myo10*, *March11* and *Ank*), which were then selected for additional bioinformatics analyses.

C3H/MOLF genetic variants in the flanking and intronic sequences were also identified for five genes (*Cdh6*, *Cdh12*, *Fbxl7*, *Ank* and *Trio*) that display reduced expression in *Gpr161*^*vl/vl*^ *Sub1*^*C/C*^ but partial or full restoration of mRNA levels in *Gpr161*^*vl/vl*^ *Sub1*^*M/M*^. The transcription start sites (TSSs) were first determined by searching Genome browser (GRCm18/mm10). Using the same query site mentioned above, the 5kb upstream promoter regions for all five genes were then searched (TSS location: *Cdh6*: 13173639; *Cdh12*: 21111452; *Fbxl7*: 26895564; *Ank*: 27466677 and *Trio*: 28025848). A total of 88 SNPs and 15 Indels were identified for three of the five genes, *Cdh6*, *Cdh12* and *Ank*. Because no variations were identified within the 5 kb upstream region for *Fbxl7* and *Trio*, the search was extended to 50kb upstream promoter region for *Fbxl7*. For *Trio*, both upstream and downstream 50kb sequences as well as the intronic region were searched. 14 SNPs and 12 Indels were identified for the two genes (S5 Table). In total 129 genetic variants were identified for additional transcription factor binding prediction analysis.

## Supporting Information

S1 Fig Mating strategy for the *Modvl4* congenic and subcongenic mice. (TIF)

S2 Fig Individual eye phenotypes in the adult (related to Fig 1). (TIF)

S3 Fig Modeling the structural context of the V201A mutation in *Ank*. (TIF)

S1 Table Phenotypes for individual E16.5 lens. (TIF)

S2 Table *Modvl4*^MOLF^ does not rescue *Gpr161*^*vl/vl*^ lethality. (TIF)

S3 Table *Modvl4* subcongenic background does not rescue *Gpr161*^*vl/vl*^ lethality. (TIF)

S4 Table Exonic SNPs and Indels within the 15 Mb minimal interval. (XLSX)

S5 Table Non-coding SNPs and Indels for *Cdh6*, *Cdh12*, *Fbxl7*, *Ank* and *Trio*. (XLSX)

S1 File Figure legends and additional references for S1, S2 and S3 Figs. (DOC)
