# Supplementary figures and images for: Congenital Cataract in Gpr161vl/vl Mice Is Modified by Proximal Chromosome 15
Source: PLoS One. 2017 Jan 30;12(1):e0170724. doi: 10.1371/journal.pone.0170724 (PMC5279759; doi:10.1371/journal.pone.0170724)

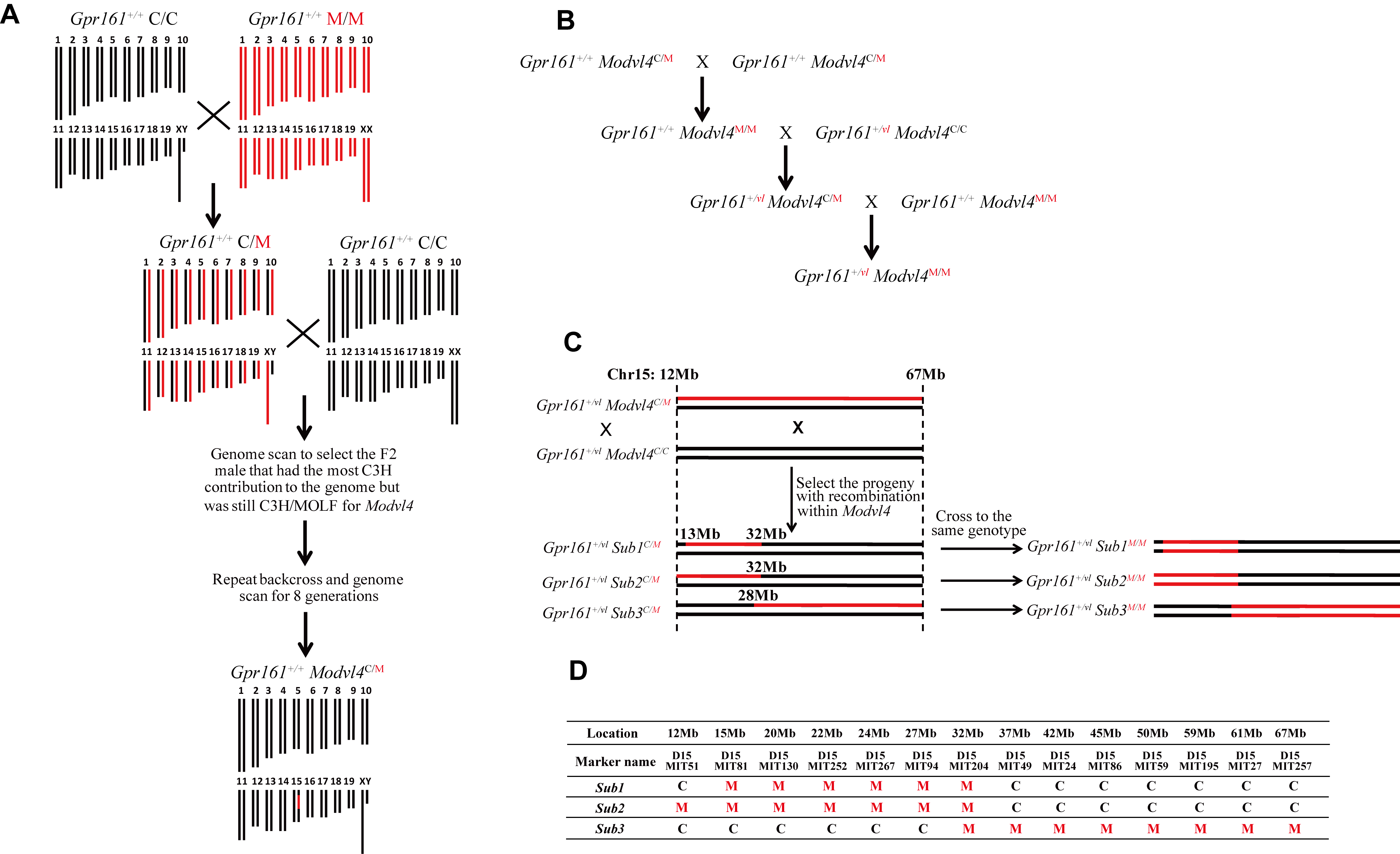

Supplement: S1 Fig — (TIF) [file pone.0170724.s001.tif]

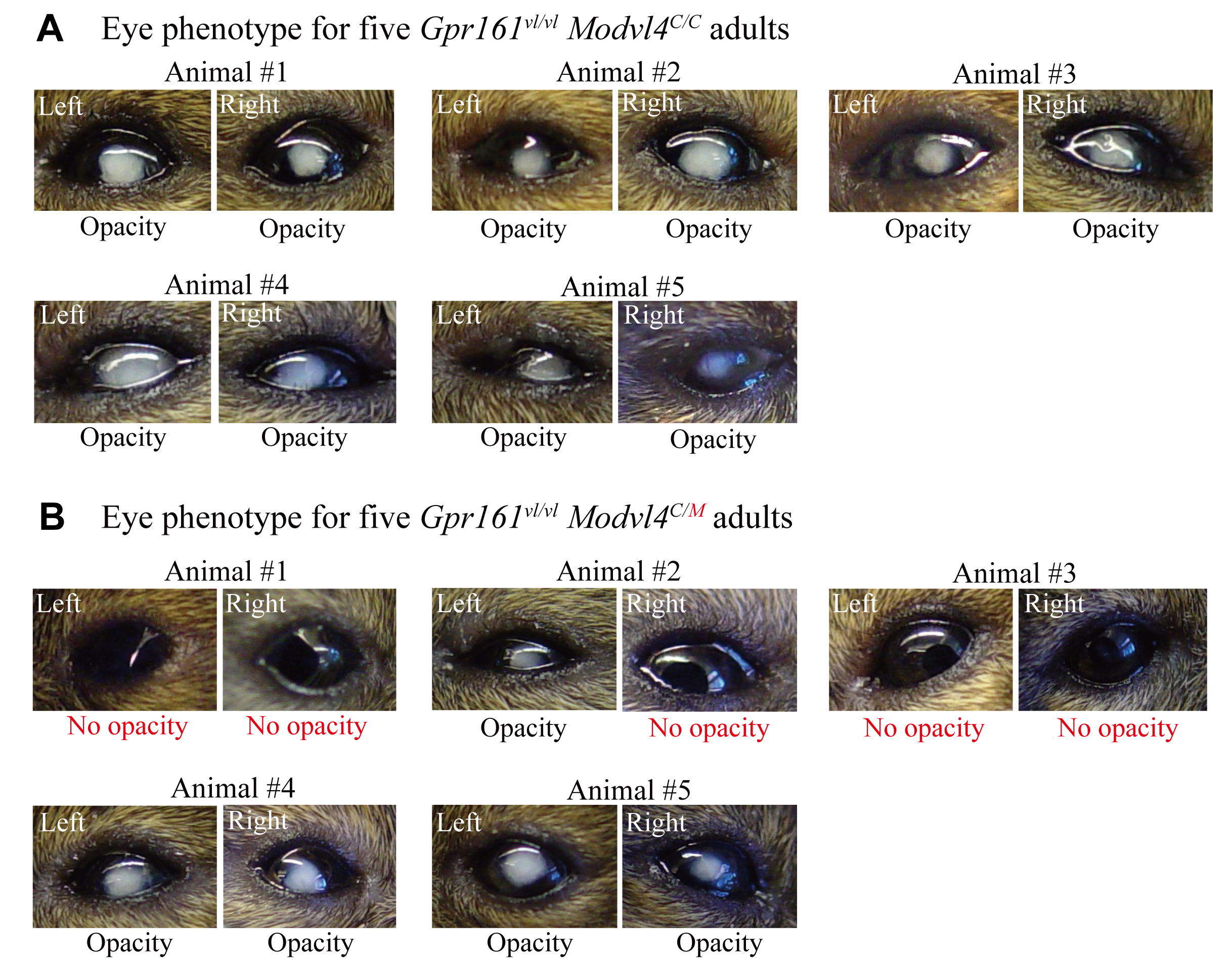

Supplement: S2 Fig — (TIF) [file pone.0170724.s002.tif]

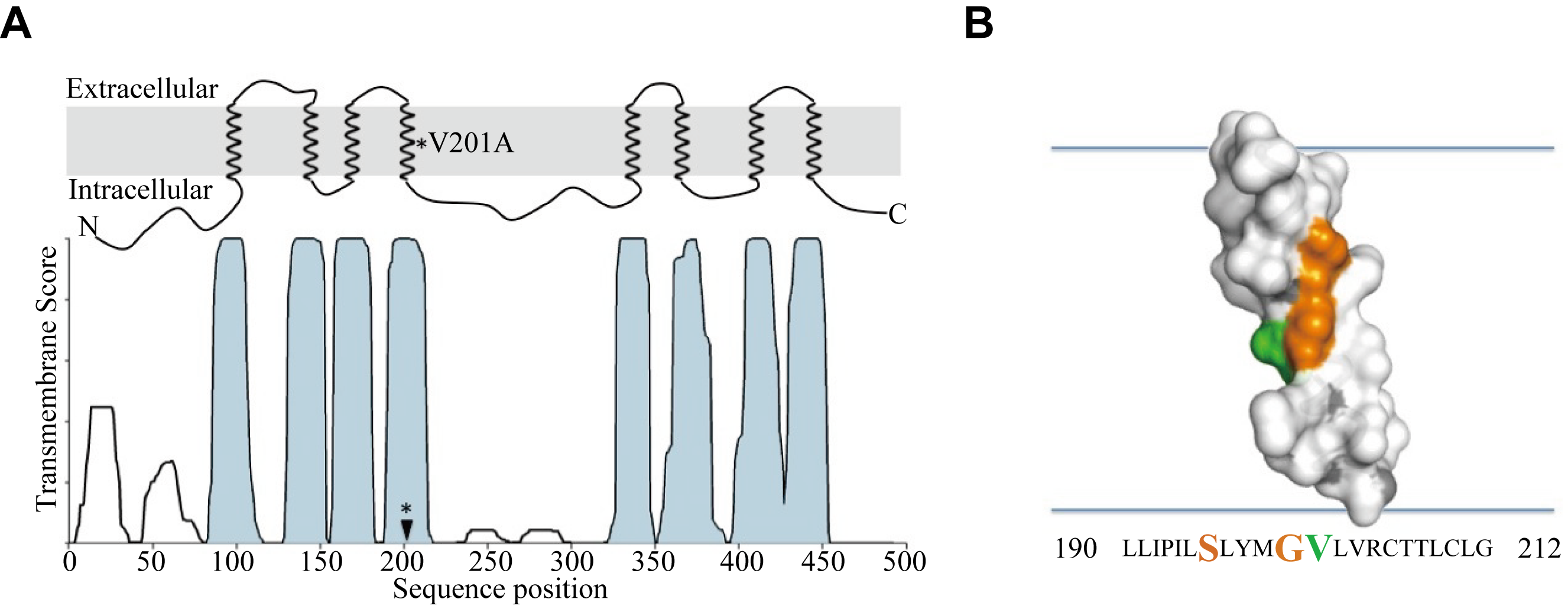

Supplement: S3 Fig — (TIF) [file pone.0170724.s003.tif]

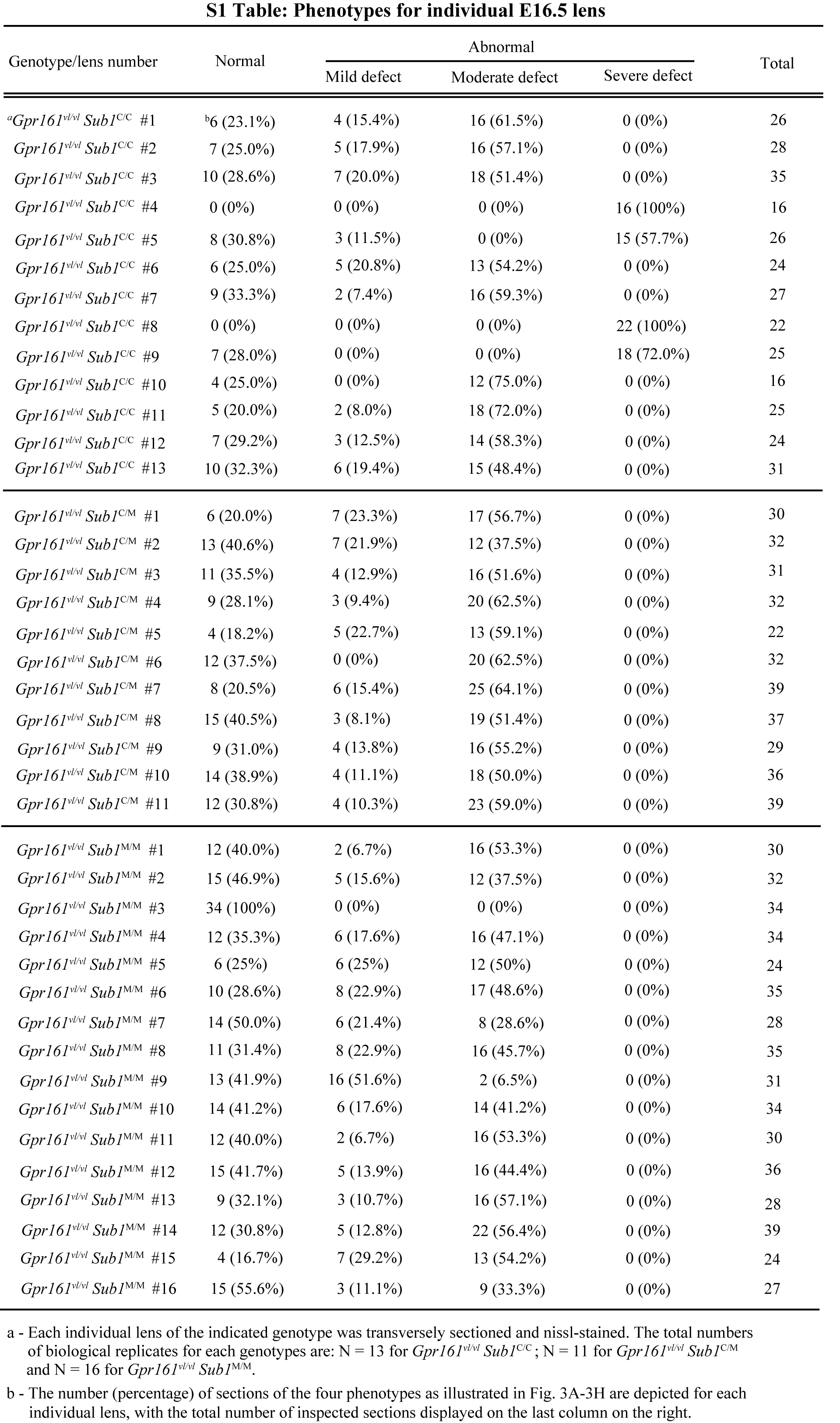

Supplement: S1 Table — (TIF) [file pone.0170724.s004.tif]

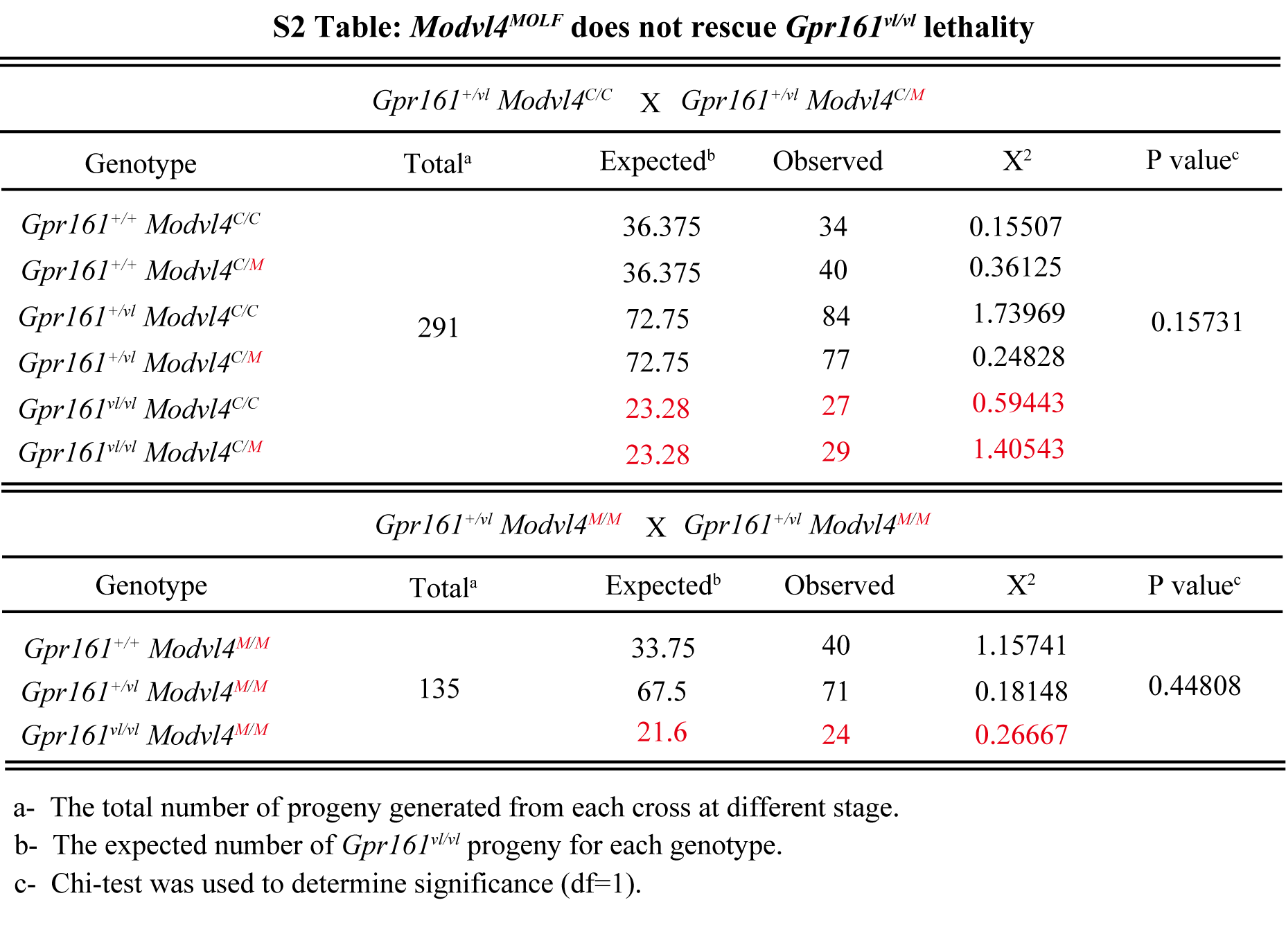

Supplement: S2 Table — (TIF) [file pone.0170724.s005.tif]

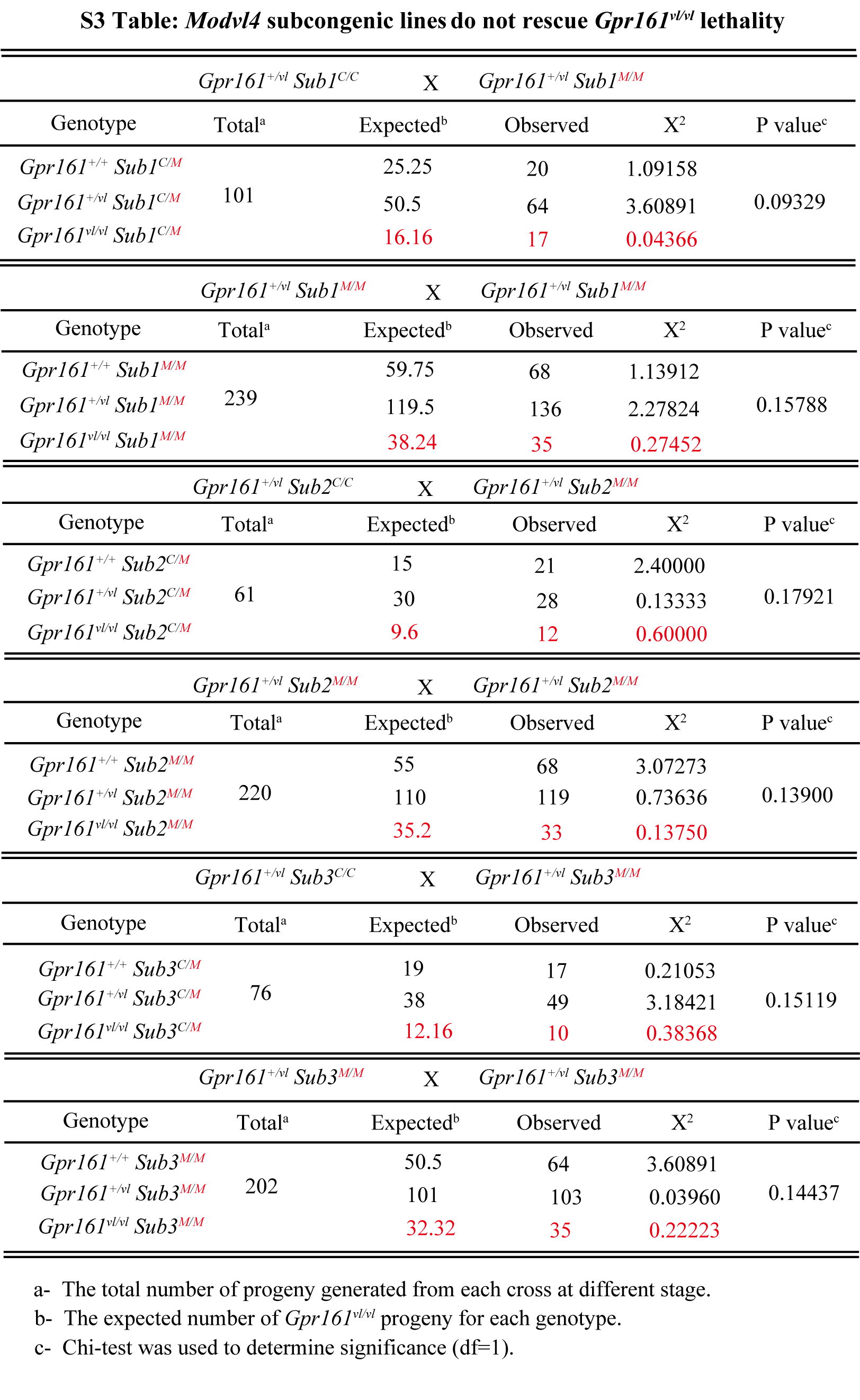

Supplement: S3 Table — (TIF) [file pone.0170724.s006.tif]
