# Supplementary material for: Congenital Cataract in Gpr161vl/vl Mice Is Modified by Proximal Chromosome 15
Source: PLoS One. 2017 Jan 30;12(1):e0170724. doi: 10.1371/journal.pone.0170724 (PMC5279759; doi:10.1371/journal.pone.0170724)
Supplement: S1 File — (DOC) [file pone.0170724.s009.doc]

**S1 File. Figure legends and additional references for S1, S2 and S3 Figs**

**S1 Fig. Mating strategy for the *Modvl4* congenic and subcongenic mice.**

(A) To generate the *Modvl4*^MOLF^ congenic, C3H/HeSnJ mice (denoted as black) were crossed to MOLF/EiJ (denoted as red), and then F1 heterozygotes were backcrossed to C3H/HeSnJ mice. Male progeny that had the most C3H contribution for the genome but were still C3H/MOLF for *Modvl4* 95% CI were selected for each subsequent generation. After 8 generations of backcrossing, *Gpr161^+/+^Modvl4^C/M^* mice were generated. (B) To cross the *Gpr161^vl^* mutation onto the *Modvl4^MOLF^* congenic background, the following matings were performed. *Gpr161^+/+^Modvl4^C/M^* were crossed to each other to generate *Gpr161^+/+^Modvl4^M/M^* congenic mice, which were then crossed to *Gpr161^+/vl^Modvl4^C/C^* to generate *Gpr161^+/vl^Modvl4^C/M^* mice. *Gpr161^+/vl^Modvl4^C/M^* mice were then crossed to *Gpr161^+/+^Modvl4^M/M^* mice to generate *Gpr161^+/vl^Modvl4^M/M^* progeny. (C) To generate the subcongenic mice, *Modvl4^C/M^* congenic was crossed to C3H isogenic mice. Progeny were genotyped for 14 SSLP markers across *Modvl4* 95% CI. In total three animals (*Sub1*, *2* and *3*) were found to contain a recombination event within *Modvl4*^MOLF^, leading to a smaller MOLF contribution than the *Modvl4^C/M^* congenic. Each animal was further crossed to C3H to generate enough progeny of the same genotype. Progeny were then crossed to each other to generate subcongenic M/M mice. (D) The name and approximate location of the 14 SSLP markers used in (C) are illustrated in the top two rows while the genotype for each of the three subcongenic lines is illustrated in the bottom three rows.

Abbreviations: C: C3H/HeSnJ; M: MOLF/EiJ; *Gpr161^vl^*: Gpr161 vacuolated lens allele; *Modvl4^C/M^*: congenic mice that are C/M in *Modvl4* interval and are C/C for all other parts of genome; X: cross to. Mb: megabases from the 5’ end of the chromosome.

**S2 Fig. Individual eye phenotypes in the adult (related to Fig 1)**

Left and right eyes from (A) five *Gpr161^vl/vl^Modvl4^C/C^* and (B) five *Gpr161^vl/vl^Modvl4^C/M^* adult mice were inspected and pictured. Phenotypes are recorded as presence or absence of opacity in the eye, and are labeled below each image. Note that in *Gpr161^vl/vl^Modvl4^C/C^* background, all ten eyes display opacity while in *Gpr161^vl/vl^Modvl4^C/M^* background, 5 out of the ten eyes display no opacity and are grossly normal.

**S3 Fig. Modeling the structural context of the V201A mutation in *Ank*.**

(A) Ank protein is predicted to contain 8 membrane-spanning regions and the Val/Ala change sits in the middle of transmembrane helix 4 (asterisk). The predicted intracellular, extracellular topology and sequence location of membrane spanning helices (highlighted in blue – plot underneath) were determined using the TMHMM server . (B) A structural model of transmembrane helix 4 indicates that position 201 (green) is adjacent to a groove formed by Ser 196 and Gly 200 (orange). Such grooves formed by small amino acids are known to mediate helix-helix interactions in the membrane and the Val/Ala change at position 201 could influence the structure of Ank or its interaction with other membrane-spanning helical proteins.

**References**

1. Krogh A, Larsson B, von Heijne G, Sonnhammer EL. Predicting transmembrane protein topology with a hidden Markov model: application to complete genomes. Journal of molecular biology. 2001;305(3):567-80. doi: 10.1006/jmbi.2000.4315. PubMed PMID: 11152613.

2. Senes A, Gerstein M, Engelman DM. Statistical analysis of amino acid patterns in transmembrane helices: the GxxxG motif occurs frequently and in association with beta-branched residues at neighboring positions. Journal of molecular biology. 2000;296(3):921-36. doi: 10.1006/jmbi.1999.3488. PubMed PMID: 10677292.

3. Walters RF, DeGrado WF. Helix-packing motifs in membrane proteins. Proceedings of the National Academy of Sciences of the United States of America. 2006;103(37):13658-63. doi: 10.1073/pnas.0605878103. PubMed PMID: 16954199; PubMed Central PMCID: PMC1564267.

4. Zhang SQ, Kulp DW, Schramm CA, Mravic M, Samish I, DeGrado WF. The membrane- and soluble-protein helix-helix interactome: similar geometry via different interactions. Structure. 2015;23(3):527-41. doi: 10.1016/j.str.2015.01.009. PubMed PMID: 25703378; PubMed Central PMCID: PMC4351763.
